# Imaging the L-Type Amino Acid Transporter-1 (LAT1) with Zr-89 ImmunoPET

**DOI:** 10.1371/journal.pone.0077476

**Published:** 2013-10-15

**Authors:** Oluwatayo F. Ikotun, Bernadette V. Marquez, Chaofeng Huang, Kazue Masuko, Miyamoto Daiji, Takashi Masuko, Jonathan McConathy, Suzanne E. Lapi

**Affiliations:** 1 Department of Radiology, Washington University School of Medicine, St. Louis, Missouri, United States of America; 2 Cell Biology Laboratory, Department of Pharmaceutical Sciences, School of Pharmacy, Kinki University, Osaka, Japan; Genentech, United States of America

## Abstract

The L-type amino acid transporter-1 (LAT1, SLC7A5) is upregulated in a wide range of human cancers, positively correlated with the biological aggressiveness of tumors, and a promising target for both imaging and therapy. Radiolabeled amino acids such as *O*-(2-[^18^F]fluoroethyl)-L-tyrosine (FET) that are transport substrates for system L amino acid transporters including LAT1 have met limited success for oncologic imaging outside of the brain, and thus new strategies are needed for imaging LAT1 in systemic cancers. Here, we describe the development and biological evaluation of a novel zirconium-89 labeled antibody, [^89^Zr]DFO-Ab2, targeting the extracellular domain of LAT1 in a preclinical model of colorectal cancer. This tracer demonstrated specificity for LAT1 *in vitro* and *in vivo* with excellent tumor imaging properties in mice with xenograft tumors. PET imaging studies showed high tumor uptake, with optimal tumor-to-non target contrast achieved at 7 days post administration. Biodistribution studies demonstrated tumor uptake of 10.5 ± 1.8 percent injected dose per gram (%ID/g) at 7 days with a tumor to muscle ratio of 13 to 1. In contrast, the peak tumor uptake of the radiolabeled amino acid [^18^F]FET was 4.4 ± 0.5 %ID/g at 30 min after injection with a tumor to muscle ratio of 1.4 to 1. Blocking studies with unlabeled anti-LAT1 antibody demonstrated a 55% reduction of [^89^Zr]DFO-Ab2 accumulation in the tumor at 7 days. These results are the first report of direct PET imaging of LAT1 and demonstrate the potential of immunoPET agents for imaging specific amino acid transporters.

## Introduction

**Figure 1:**
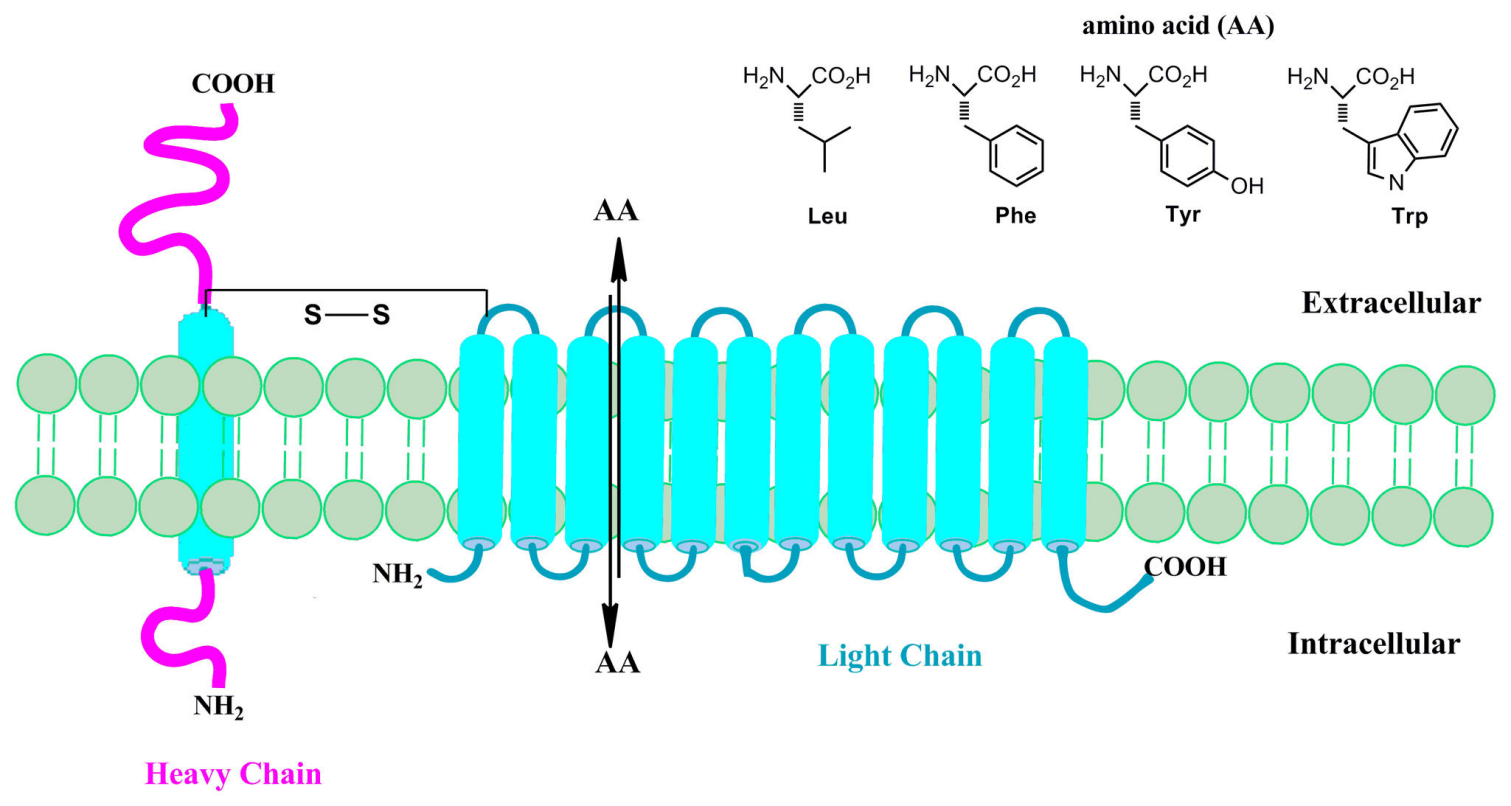
An illustration of the transmembrane L-type amino acid transporter-1 (LAT1) which forms a functional heterodimer with the 4Fhc heavy chain glycoprotein and is responsible for the transport of amino acids with large neutral side chains through an exchange mechanism.

Alterations in metabolism and nutrient transport are hallmarks of neoplastic cells and represent important targets for molecular imaging. A prototypic example is the Warburg effect, the upregulation of the conversion of glucose to lactate via glycolysis, which occurs in many cancers and is the basis of clinical oncologic positron emission tomography (PET) imaging with the glucose analogue, 2-deoxy-2-[^18^F]fluoro-D-glucose (FDG). Amino acid transport is also increased in many cancers and has been effectively targeted with radiolabeled amino acid substrates for PET and single photon emission computed tomography (SPECT) imaging. The majority of amino acid-based tracer development has focused on system L amino acid transport, in particular, the solute carrier (SLC) protein L-type amino acid transporter-1 (LAT1, SLC7A5) which preferentially mediates the sodium-independent cellular transport of amino acids with large neutral amino acid side chains such as leucine, phenylalanine, methionine, tyrosine, and tryptophan [1-3]. The functional LAT1 transporter, shown in Figure 1, is a disulfide linked heterodimeric transmemberane protein comprised of two subunits: a heavy chain glycoprotein (4Fhc; SLC3A2), and the non-glycosylated LAT1 light chain protein with 12-transmembrane domains [4,5]. In addition to providing amino acids for protein synthesis and other metabolic pathways, LAT1 is involved in promoting cellular growth and proliferation, angiogenesis, and mTOR pathway signaling [6-8]. Higher levels of LAT1 are positively correlated with increased biological aggressiveness and higher mortality in a range of human cancers including gliomas, breast, lung, prostate, and ovarian cancers and is thus a promising target for tumor imaging and therapy [2,9-12]. 

Radiolabeled L substrates including 6-[^18^F]fluoro-L-3,4-dihydroxy-phenylalanine ([^18^F]FDOPA), L-methyl-^11^C-methionine ([^11^C]MET), *O*-(2-[^18^F]fluoroethyl)-L-tyrosine (FET), 3-[^123^F]fluoro-α-methyl-L-tyrosine (FAMT), and 3-[^123^I]iodo-α-methyl-L-tyrosine (IMT) have been employed extensively for oncologic imaging in patients; and are particularly effective for brain tumor imaging as system L transport is up-regulated in glioma cells and is active at the intact blood-brain barrier [13-17]. However, these substrates have generally been inferior to FDG for imaging tumors outside of the brain, primarily due to lower sensitivity for tumor detection [18]. The limited sensitivity is a consequence of the bi-directional nature of LAT1 and other system L transporters, which mediate both influx and efflux of substrates from cells through an exchange mechanism of transport. Thus, LAT1 cannot directly concentrate substrates, leading to relatively low tumor to tissue ratios. Additionally, the selectivity for radiolabeled amino acids targeting LAT1 over other system L transporters (LAT2, LAT3 and LAT4) is not typically complete as demonstrated through *in vitro* studies using cells with experimentally reduced levels of LAT1 [19,20]. This lack of selectivity is important because other system L transporters do not appear to be overexpressed to the same degree as LAT1 in human cancers [21]. Additionally, it is not clear if structural modifications of existing system L transport substrates can substantially improve selectivity for LAT1 over other system L transporters. Thus, the substrate-based approach for imaging system L transport has inherent limitations, and improved PET tracers targeting LAT1 outside of the brain will require a different strategy.

Towards this end, we have developed an immunoPET strategy for imaging LAT1 that overcomes many of the limitations of substrate-based tracers. Monoclonal antibodies that bind to cell surface targets are versatile and useful scaffolds for molecular imaging and can achieve high specificity and binding affinity to tumor antigens of interest. This work takes advantage of a monoclonal antibody recently developed by Masuko and colleagues that directly and specifically target the extracellular domain of native human LAT-1 protein [1,22,23]. In this report, we describe the radiolabeling method and the *in vitro* and *in vivo* tumor imaging properties of a novel ^89^Zr-labeled anti-LAT1 antibody, [^89^Zr]DFO-Ab2, in the HCT116 human colorectal cancer model. 

## Materials and Methods

### Production and characterization Anti-human LAT1 mAb

Production and characterization of similar anti-human LAT1 and anti-human CD98 heavy chain (CD98hc) mAb were reported by Ohno et al [1]. Ab2 was produced against HeLa cells highly expressing well-defined LAT1 proteins disulfide-linked to CD98 heavy chain [1]. Female F344/N rats were administered subcutaneous and intraperitoneal injections (first and second immunizations) followed by a final intravenous injection of HeLa cells (3.0 x 10^7^) in each immunization at 3-week intervals. The immune spleen cells (1.0 x 10^8^) were fused with X63 mouse myeloma cells (2.5 x 10^7^) using 50% polyethylene glycol 1540 (Roche, Penzberg, Germany). After the cell fusion, hybridoma cells were selected in RPMI supplemented with hypoxanthine, aminopterin and thymidine (50 x HAT; Invitrogen). Isotypes of the selected anti-human LAT1 rat mAb (Ab2) used in this study are γ2a and κ. Ab2 strongly reacted with RH7777 rat hepatoma cells (kindly donated by Dr. Chiba, Mitsubishi Tanabe Pharma, Yokohama, Japan) expressing human LAT1-GFP and HCT-116 cancer cells [1,22]. 

### Cell Culture

Rat hepatoma cell line (RH7777) was obtained from American Type Culture Collection (ATCC; Manassas, VA) and cultured in Dulbecco's modified Eagle’s medium (DMEM; Sigma-Aldrich, St. Louis, MO) supplemented with 7% heat-inactivated fetal bovine serum (FBS; ICN Biomedicals, Aurora, OH). Human colorectal cancer cell line, HCT-116 (ATCC), was grown in Iscove’s media supplemented with 10 % FBS and 10 µg/mL gentamicin (Life Technologies, Carlsbad, CA). Cells were maintained at 37 °C in 5 % CO_2_, and 90 % humidity. After 4 passages, vials of cells were frozen and stored in liquid nitrogen for future use. Fresh vials of cells were periodically thawed and used for *in vitro* experiments to ensure that changes to cells did not occur over time or with passages in culture. For xenograft models, a fresh vial of cells was thawed 10 - 14 days before tumor implantation.

### Flow cytometry

For cell-surface staining, cells (3.0 x 10^5^) in 50 µl of PBS containing 1% BSA were mixed with purified Ab2 (50 µL) diluted to 10 µg/ml in 1% BSA-PBS and incubated for 1 h at 4°C. Cells were washed three times with PBS, and were incubated with 50 µl of 1:200 diluted donkey anti-rat IgG (H+L) antibodies labeled with phycoerythrin (Jackson ImmunoResearch, West Grove, PA) at 4 °C for 30 min. After three washes with PBS, cells were suspended in PBS containing 0.2% BSA, and analyzed with an Accuri C6 flow cytometer (Tomy Digital Biology, Tokyo, Japan). 

### Ab2 Antibody and Rat IgG Conjugation and Radiolabeling

The anti-LAT 1 antibody, Ab2 (1 - 4.5 mg/mL), and control polyclonal rat IgG from rat serum (Sigma Aldrich, St. Louis, MO) were conjugated with the chelator desferoxamine (DFO) in 0.1 M sodium carbonate (NaHCO_3_) buffer (pH 9) following established methods [24-27]. A 5 mg/ml stock solution of *p*-isothiocynatobenzyl-desferroxamine (DFO-Bz-NCS) was made in DMSO. To each antibody, an 8 fold molar excess of the DFO chelator, and 0.1 M NaHCO_3_ buffer was added to bring the reaction volume to 150 µL and ensure the reaction pH was maintained at 9. The reaction solution was incubated at 37 °C for 1 h, and unreacted DFO was removed by size exclusion chromatography on a 40 kDa cutoff Zeba^TM^ spin desalting column (Thermo Fisher Scientific, Rockford, IL). The protein concentration of the resultant DFO functionalized antibodies were determined by bicinchoninic acid (BCA) assay (Thermo Scientific, Rockford, IL). ^89^Zr was produced via the ^89^Y(p,n)^89^Zr reaction on a CS-15 cyclotron (Cyclotron Corporation, Berkeley, CA) at the Washington University in St. Louis Cyclotron Facility and purified as previously reported [28]. For radiolabeling, 18 - 111 MBq (0.5 - 3 mCi) of ^89^Zr-oxalate solution (pH ≤ 1) was neutralized to pH 6.9 - 7.2 by first adding an equivalent volume of 0.2 M HEPES (pH 7), followed by slow addition of 2M Na_2_CO_3_. The DFO functionalized mAb was added to ^89^Zr-oxalate solution and incubated at 37 °C for 1 hour while shaking. A 5 µL aliquot was removed and challenged with 5 µL of 50 mM diethylenetriaminepentaacetic acid (DTPA) to assay for unreacted and non-specifically bound ^89^Zr. Radiochemical purity was determined by ITLC using 50 mM DTPA as the mobile phase. Antibodies with radiochemical purity of ≥ 95 % were used without further purification.

### Synthesis of [^18^F]FET

The production of [^18^F]FET was performed using the Eckert and Ziegler Modular Lab based on a previously reported automated procedure with a solid-phase extraction purification [29]. [^18^F]fluoride was produced from [^18^O]H_2_O via the ^18^O(p,n)^18^F reaction on the RDS 111 cyclotron (Siemens, TN). [^18^F]FET was formulated in 10 % ethanol in saline for studies. The specific activity was calculated to be greater than 37 GBq (1 Ci) per µmol (n=4).

### Immunoreactivity Assay

The immunoreactive fraction of [^89^Zr]DFO-Ab2 was determined using a modification of the radioactive cellular-binding assay described by Lindmo et al.[30]. Briefly, HCT116 cells were suspended in microcentrifuge tubes at concentrations of 3, 2.4, 1.8, 1.5, 1.2, 0.9, 0.6, and 0.3 x 10^6^ cells/mL in 500 µL PBS (pH 7.4). Aliquots of [^89^Zr]DFO-Ab2 ( 50 µL of a stock solution of 0.11 MBq (3 µCi) in 30 mL of 1 % bovine serum albumin (BSA); 2,000 cpm (1.3 ng) of radiolabeled mAb were added to each tube (n = 3) for a final volume of 550 µL. Samples were incubated at room temperature for 1 h while shaking. After incubation, cells were pelleted by centrifugation, resuspended and washed twice with ice-cold PBS. The supernatant was removed and cell associated activity counted on a γ–counter. The background corrected count data was compared with the total activity added. The (total/bound) activity was plotted against the inverse cell concentration (1/[normalized cell concentration]). The immunoreactive fraction was determined by linear regression analysis of the plot, and calculated from the inverse of the *y*-intercept. 

### In vitro Studies

#### Saturation receptor-binding assay

The dissociation constant (K_d_) for [^89^Zr]DFO-Ab2 was measured using a radioligand saturation receptor-binding assay. Approximately 1 x 10^5^ of LAT-1 expressing HCT116 cells were seeded in a 48 well plate and allowed 48 hours to achieve ~50 % confluency. The adherent cells were incubated with increasing concentrations of [^89^Zr]DFO-Ab2 (0 - 450 nmol/L) in media containing 10 % FBS to eliminate non-specific binding, at a total volume of 100 µL for 2 hours at 37 °C. Unbound radioactivity was removed and the cells washed three times with phosphate-buffered saline (PBS), pH 7.4. Cells were removed by trypsinization and cell associated radiation counted on a γ–counter. The K_d_ was calculated by fitting a plot of cell-bound [^89^Zr]DFO-Ab2 (nmol) vs. the concentration of unbound radioligand (nmol/L) to a one-site saturation binding model using Prism® Ver. 5.0 software (GraphPad Software, San Diego, CA, USA). This model assumes that the amount of nonspecific binding is proportional to the concentration of radioligand. 

#### Rate of Internalization

HCT116 cells were seeded in 24 well plates at 2.4 x 10^5^ cells per well and were allowed 24 h to adhere. Fresh complete media was added and cells incubated with 6 µg/mL of [^89^Zr]DFO-Ab2 (2 µL of mAb in 200 µL of complete media) for varying time points: 0.2, 0.5, 1, 2, 4, 6, and 24 h at 37 °C. The media was removed and cells were washed twice with ice-cold PBS to remove unbound [^89^Zr]DFO-Ab2. Thereafter, cells were trypsinized, and treated with 300 µL of 0.1 M sodium citrate (pH 2) at 37 °C for 5 min. After incubation, cells were pelleted by centrifugation, the amount of cell surface-bound activity, and intracellular radioactivity was determined by measuring the radioactivity of the supernatants and the cell pellets, respectively, in a γ-counter. The relative percent bound activity for surface bound and internalized activity was plotted over time. 

### HCT116 Xenograft Model

All animal experiments were performed according to the guidelines of the Institutional Animal Care and Use Committee (IACUC) and the Washington University Animal Studies Committee specifically approved this study. Animals were provided food and water *ad libitum* prior to imaging studies. Athymic nude *nu/nu* 7 week old male mice were purchased from National Cancer Institute (Frederick, MD) and housed under pathogen-free conditions in an approved facility. Animals (24-28 g) were implanted subcutaneously (shoulder) with 2.0 x 10^6^ cells of HCT116 cells suspended in 100 µl of saline, and developed tumors averaging 150-200 mm^3^ approximately 14 days after implantation. 

### Biodistribution Studies

Biodistribution studies were conducted to evaluate the uptake of [^89^Zr]DFO-Ab2 in HCT116 tumor bearing mice. Mice were randomized (n = 3 for each group) and warmed gently under a heat lamp for approximately 5 minutes prior to tail vein administration of radiotracer. Mice received [^89^Zr]DFO-Ab2 (3.1 - 3.3 MBq [83 - 88 µCi], 15 - 16 µg of mAb) or [^89^Zr]DFO-IgG (1.0 - 2.3 MBq [28 - 57 µCi], 21 - 48 µg of Ab) as a non-specific control, in 100 µL 0.9 % sterile saline. Animals were euthanized by cervical dislocation at 3 or 7 days post administration. A separate set of animals ( n = 4- 5) were injected with 1.8 - 5.6 MBq (50-150 µCi) of [^18^F]FET in 100 µL of 10% ethanol in normal saline via tail vein administration, and were sacrificed at 5, 30, and 60 minutes post administration. Organs of interest (including the tumor) were harvested and weighed, and radioactivity measured on a gamma-counter. Syringes of the formulation injected into the animals were used as standards. Data were background- and decay-corrected, and the percent injected dose per gram (%ID/g) for each tissue sample calculated by normalization to the total activity injected. 

Blocking studies were performed to investigate the specificity of [^89^Zr]DFO-Ab2 for LAT1 *in vivo*. Non-radiolabeled Ab2 antibody (0.8 mg/mouse) was pre-administered intravenously (tail vein) 1 hour prior to [^89^Zr]DFO-Ab2 injection and biodistribution studies were conducted as described above.

### Small-Animal ImmunoPET Imaging

Mice were anaesthetized with 1-2 % isoflurane, injected intravenously with 3.1 - 3.3 MBq (88 - 98 µCi, 22-25 µg) of [^89^Zr]DFO-Ab2 ( n = 3) and imaged on an Inveon small animal PET/CT scanner (Siemens Medical Solutions) at 72 and 168 h post injection. Static images were collected for 30 minutes for the ^89^Zr PET imaging studies. A separate set of rodents (n= 4) received 150 µCi of [^18^F]FET intravenously followed by 60 minute dynamic PET scans. Images were reconstructed with the Maximum A Posteriory Probability (MAP) algorithm followed by co-registration with the Inveon Research Workstation image display software (Siemens Medical Solutions, Knoxville, TN). Regions of interest (ROI) were selected from PET images using CT anatomical guidelines and the associated activity measured with Inveon Research Workstation software. Maximum Standard uptake values (SUV) were determined as Bq/cc x animal weight (g)/ injected dose (Bq).

### Immunohistochemistry

Excised tumor masses were fixed in 4 % PFA in PBS and 5-7 µm sections were used for immunostaining. Deparaffined sections were heated at 60 °C for 20 min and hydrated in xylene and graded alcohol. Tissue sections were then coated with normal blocking serum (1:50) for 30 min to reduce nonspecific binding. Tissue sections were incubated with a commercially available anti-human LAT1 mAb at 1:100 (TransGenic Inc., Chuo-ku, Kobe) overnight followed by washing with tris-buffered saline with tween (TBST). Slides were incubated with biotinylated rabbit anti-rat IgG (Vector Laboratories, Bulingame, CA) diluted to 1:200 in PBS for 30 min. After three washes with TBST, samples were treated with ABC reagent (Vector Laboratories) at 1:100 for 30 min. Tissues were stained with alkaline phosphatase substrate kit (Vector Laboratories) for 2 min, and then color development was stopped by placing slides in water. Counterstaining was conducted with nuclear fast red for 30 min, followed by dehydration in ethanol. The location of antibody-defined components were observed under a Zeiss Axiophot microscope (Zeiss, Thornwood, NY) and photographed. 

### Statistical Analysis

Data were analyzed with the student *t* test when comparing two groups or one way ANOVA with Bonferroni post-test analysis for multiple comparisons. Differences with 95 % confidence level (P < 0.05) were considered to be statistically significant. 

## Results

### Conjugation and Radiolabeling

The anti-LAT1 antibody, Ab2, was modified with the acyclic bifunctional chelator, Df-Bz-NCS at an 8:1 molar excess of chelate to protein in sodium carbonate buffer (pH 9). Unreacted Df-Bz-NCS was removed by size exclusion chromatography, and the concentration of functionalized anti-LAT1 antibody quantified by BCA assay. Using this method, we achieved ≥ 90 % recovery of our antibodies; for DFO-Ab2 the post purification concentration was 4.2 mg/mL and 2.5 mg/mL for non-specific IgG. Post modification analysis on fast protein liquid chromatography (FPLC) revealed the antibody maintained its’ integrity with less than 5 % aggregation products observed. The DFO functionalized antibodies were radiolabeled using ^89^Zr-oxalate. [^89^Zr]DFO-Ab2 was radiolabeled at a specific activity of 5 mCi/mg and the control IgG was radiolabeled at 1 mCi/mg. Under these conditions ≥ 95% of the added activity was associated with the antibody as determined by radioTLC. The challenge radiochemical purity as assessed by DTPA challenge was 97.6 ± 1.2 %, and the radiolabeled mAb was used without further purification. The resulting specific activities were 189.1 ± 10 MBq/mg (5.14 ± 0.33 mCi/mg) for [^89^Zr]DFO-Ab2, and 48.8 ± 8.1 MBq/mg (1.32 ± 0.22 mCi/mg) for [^89^Zr]DFO-IgG. 

### In Vitro characterization

**Figure 2:**
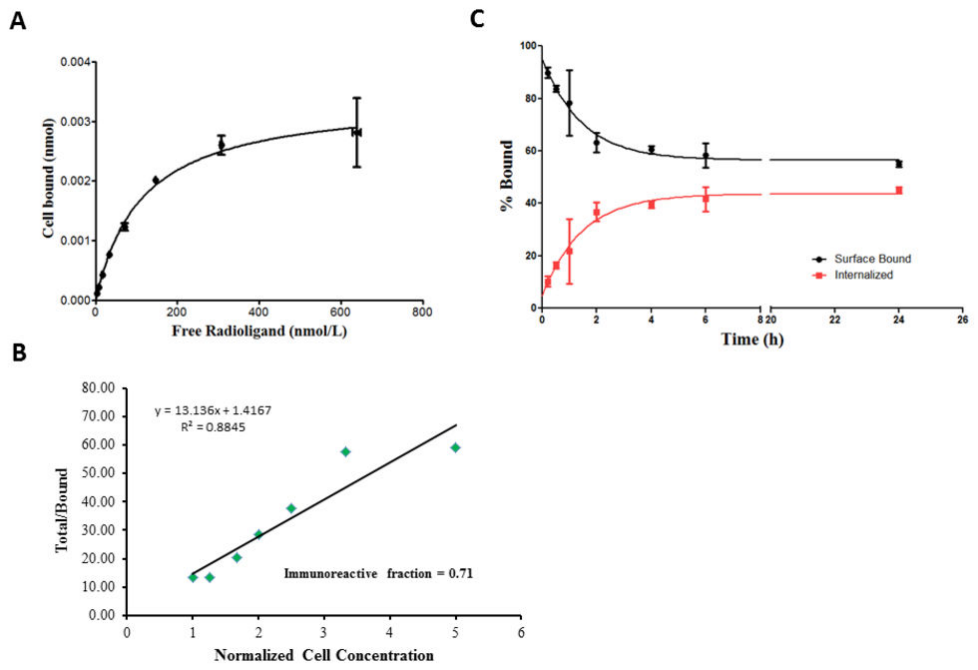
*In* *vitro* experiments in LAT-1 expressing HCT-116 cell line. (**A**) Receptor saturation using varying concentrations of the radiolabeled antibody, [^89^Zr]DFO-Ab2. (**B**) Immunoreactivity assay plot of the (total/bound) activity *versus* (1/[normalized cell concentration]) of [^89^Zr]DFO-Ab2, immunoreactive fraction determined by extrapolation to infinite antigen excess (1/y-intercept). (C) Surface bound and internalized cellular accumulation of the radioimmunoconjugate over time (up to 24 h).

Flow cytometry studies using rat liver RH7777 cells transfected with green fluorescent protein (GFP) fused human and mouse CD98 light chains demonstrated the specificity of the anti-LAT antibody, Ab2, for human LAT1 (shown in Figure S1). Interspecies cross reactivity was not observed, additionally, the antibody did not react with the anchoring CD98 heavy chain. [^89^Zr]DFO-Ab2 demonstrated saturable binding to LAT1-expressing HCT116 cells. The concentration at which [^89^Zr]DFO-Ab2 occupies 50 % of cell surface receptors (K_d_) was determined to be 112.5 ± 27.2 nM, (shown in Figure 2). To ascertain the purity and biological integrity of the radioconjugate, an immunoreactivity assay was conducted and the immunoreactive fraction at infinite LAT1 antigen excess was calculated as 0.79 ± 0.11. We examined the binding kinetics of [^89^Zr]DFO-Ab2 where surface bound [^89^Zr]DFO-Ab2 was internalized at 37 °C for various intervals up to 24 h as shown in Figure 2 [31]. Over-time, the surface-bound activity decreased as the internalized fraction increased. The intracellular radioactivity reached a steady-state after 4 hours, with no change in associated activity between 6 and 21 hours with approximately 40% of the activity internalized and 60% bound to the cell surface.

### Biodistribution Studies and *In Vivo* Imaging

**Figure 3:**
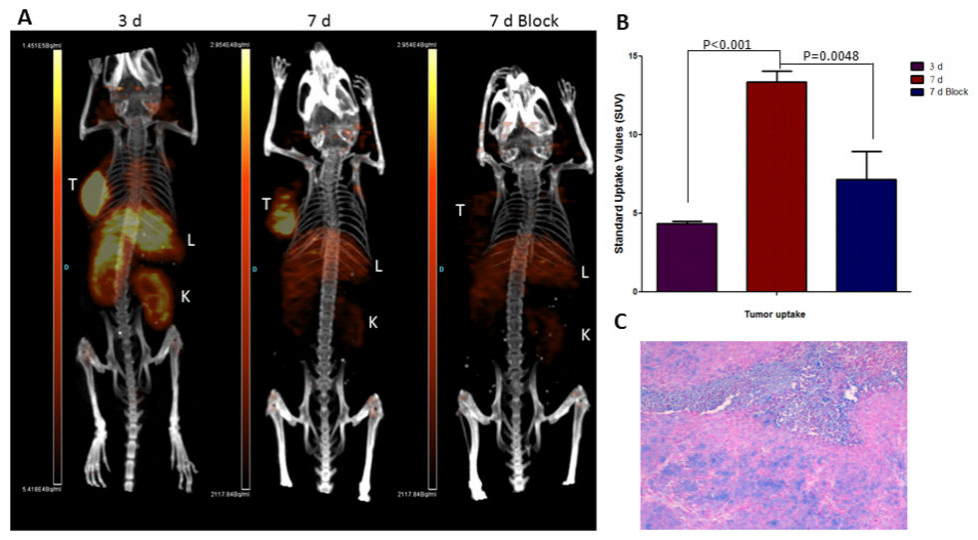
*In* *vivo* immuno-PET images of [^89^Zr]DFO-Ab2 in HCT-116 bearing xenografts. (**A**) Representative maximum intensity projections (MIP) of small animal PET/CT images at 3 and 7 days, where tumor is clearly visualized at 3 d. The tracer uptake was blocked with 800 µg of excess Ab2-LAT-1 (7 d block). (**B**) Maximum standard uptake values confirming increased tracer accumulation in tumor lesion over time; significant decrease in accumulation was observed in the presence of blocking dose. (**C**) Immunohistochemistry showing expression of LAT-1 in excised HCT-116 tumor used in this study.

We investigated the tumor and normal tissue distribution of [^89^Zr]DFO-Ab2 in a subcutaneous model of human colorectal cancer through PET and biodistribution studies conducted at 3 and 7 days after intravenous administration. Small animal PET/CT images revealed relatively high accumulation of activity in the tumor at 3 days post injection (p.i.), although blood pool, liver, and kidney activity was also relatively high at this time point. At 7 days p.i., the activity in the tumor persisted while the non-target tissue activity cleared substantially. Quantitative region-of-interest analysis of PET images revealed a 3-fold increase in tumor associated standard uptake values (SUVs), with values of 2.2 ± 0.07 observed at 3 d increasing to 3.0 ± 0.24 at 7 days (Figure 3). Immunohistochemistry staining was conducted on the excised tumor mass, and the presence of LAT1 protein was independently confirmed using a commercially available anti human LAT1 monoclonal antibody (Figure 3C).

*Ex vivo* biodistribution studies corroborated the PET data with high levels of tumor uptake observed for the [^89^Zr]DFO-Ab2 immunoPET tracer. The probe was retained in the tumor with uptake of 9.3 ± 0.84 and 10.5 ± 1.8 %ID/g at 3 and 7 d p.i., respectively. Improved contrast was achieved at later time points, when the blood-pool activity decreased from 5.6 ± 0.36 at 3 d to 3.0 ± 0.07 %ID/g at 7 d p.i. The %ID/g values for most normal tissues were substantially lower than the tumor at both time points with clearance of activity from 3 to 7 d (Table 1), a profile that is consistent with the blood clearance of radiolabeled antibodies [27,32]. The highest concentration of activity in normal tissues occurred in the liver with 7.6 ± 1.2 and 7.6 ± 0.36 %ID/g at day 3 and day 7 p.i. respectively, a distribution typical of radiolabeled antibodies. Accumulation of activity in bone may be indicative of decomplexation of ^89^Zr from the intact functionalized mAb; however the increase from 3.0 ± 0.30 to 3.9 ± 0.25 %ID/g (p = 0.0176) at 3 and 7 d respectively may be a consequence of tracer metabolism and localization of ^89^Zr species into the bone [33,34]. 

**Table 1:** Biodistribution of intravenously administered ^89^Zr-Ab2-DFO in selected organs of male Athymic *nu/nu* mice bearing subcutaneous HCT-116 tumors.

	**[^89^Zr**]**DFO-Ab2**						**Block**			**Rat IgG**					
**Organ**	**3 d (n=3**)			**7 d (n = 3**)			**7 d (n = 3**)			**3 d (n=3**)			**7 d (n=3**)		
blood	5.58	+/-	0.36	3.04	+/-	0.07	3.45	+/-	0.35	4.76	+/-	0.39	3.20	+/-	0.63
lung	3.32	+/-	0.24	2.25	+/-	0.12	2.59	+/-	0.62	2.28	+/-	0.26	2.83	+/-	1.15
liver(all)	7.64	+/-	1.24	7.63	+/-	0.36	8.56	+/-	0.82	9.77	+/-	1.30	10.38	+/-	2.15
spleen	4.26	+/-	0.48	3.88	+/-	0.15	4.73	+/-	0.78	3.56	+/-	1.20	4.85	+/-	1.20
kidney	11.15	+/-	1.70	6.99	+/-	1.04	7.33	+/-	0.20	2.63	+/-	0.31	2.28	+/-	0.15
muscle	1.14	+/-	0.11	0.81	+/-	0.05	0.88	+/-	0.06	0.61	+/-	0.07	0.55	+/-	0.11
heart	2.59	+/-	0.10	1.68	+/-	0.15	1.79	+/-	0.09	1.57	+/-	0.26	1.23	**+/-**	0.07
brain	0.25	+/-	0.04	0.14	+/-	0.03	0.16	+/-	0.03	0.18	+/-	0.05	0.14	+/-	0.03
bone	2.99	+/-	0.30	3.86	+/-	0.25	4.39	+/-	0.38	1.99	+/-	0.49	3.01	+/-	0.97
testes	2.16	+/-	0.16	1.95	+/-	0.06	2.01	+/-	0.12	1.05	+/-	0.20	0.88	+/-	0.12
pancreas	1.17	+/-	0.12	1.10	+/-	0.08	0.95	+/-	0.03	0.78	+/-	0.24	0.78	+/-	0.14
tumor	9.29	+/-	0.84	10.49	+/-	1.78	4.70	+/-	0.08	2.24	+/-	0.47	1.49	+/-	0.42
stomach	0.40	+/-	0.09	0.26	+/-	0.06	0.32	+/-	0.09	0.43	+/-	0.10	0.27	+/-	0.05
sm int	0.78	+/-	0.05	0.55	+/-	0.10	0.63	+/-	0.05	0.60	+/-	0.04	0.52	+/-	0.09
u lg int	0.80	+/-	0.22	0.51	+/-	0.08	0.57	+/-	0.14	0.57	+/-	0.10	0.51	+/-	0.13
l lg int	0.67	+/-	0.12	0.53	+/-	0.06	0.57	+/-	0.09	0.58	+/-	0.14	0.39	+/-	0.09
Data expressed in %ID/g ± SD.															

Blocking studies with pre-injection of 0.8 mg of unlabeled Ab2 were conducted in another group of mice to assess for specific binding of [^89^Zr]DFO-Ab2 *in vivo*. In this group, the tumor was poorly visualized with tumor associated activity decreasing to 4.7 ± 0.08 %ID/g at 7 d, a 55% decrease relative to the control group (p = 0.014). These experiments are in agreement with the *in vitro* data and support the specificity of [^89^Zr]DFO-Ab2 for LAT1. As an additional control, biodistribution studies were performed using ^89^Zr-labeled non-targeted polyclonal rat IgG ([^89^Zr]DFO-IgG) to assess the contribution of non-specific uptake mechanisms such as blood volume and passive diffusion. We observed superior tumor accumulation with [^89^Zr]DFO-Ab2 compared to [^89^Zr]DFO-IgG with tumor uptake of 1.5 ± 0.42 %ID/g (p = 0.0006) at 7 days p.i. These results further support the specificity of [^89^Zr]DFO-Ab2 for LAT1 *in vivo*.

**Figure 4:**
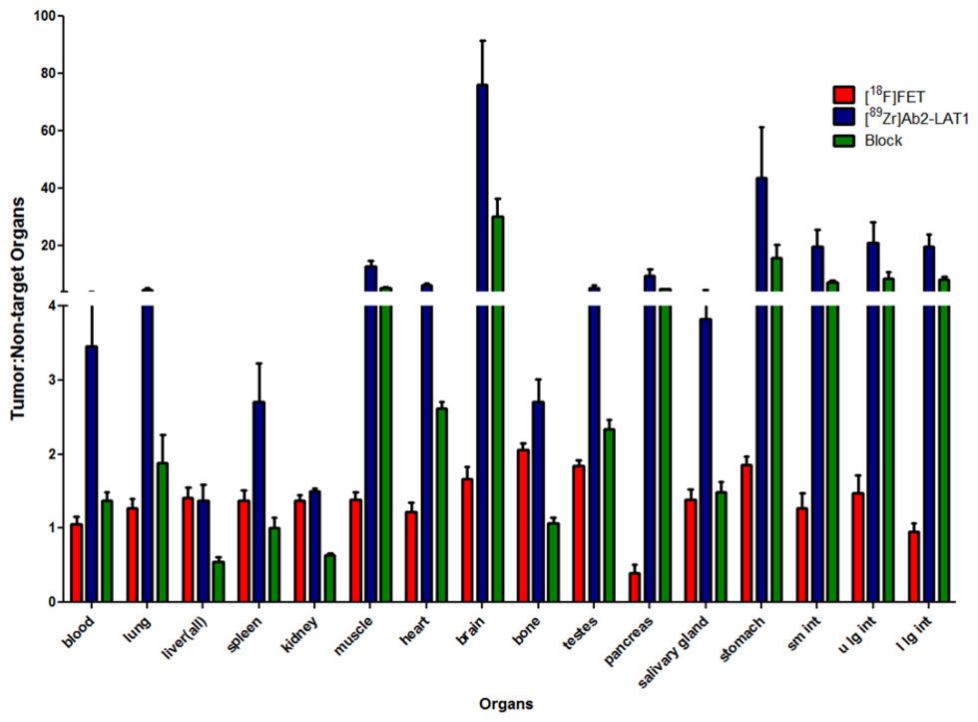
Tumor-to-organ ratios of the immunoPET tracer, [^89^Zr]DFO-Ab2 without (blue) and with blocking doses (green) of unlabeled Ab2 at 7 days post injection with comparison to the ^18^F-labeled amino acid, [^18^F]FET, at 30 minutes after intravenous administration.

To further assess the performance of [^89^Zr]DFO-Ab2, we compared the biodistribution and PET imaging properties of [^89^Zr]DFO-Ab2 with the well-established system L substrate, [^18^F]FET, in the same HCT116 tumor model. The peak uptake of [^18^F]FET occurred at 30 min p.i. with 4.4 ± 0.51 %ID/g in the tumor as shown in Table 2. The absolute amount of tumor uptake observed with [^18^F]FET as well as the tumor to normal tissue ratios were significantly lower than those observed with [^89^Zr]DFO-Ab2 as illustrated in Figure 4. These results with [^18^F]FET are in keeping with previous reports in preclinical and human tumor imaging studies for neoplasms outside of the brain [18]. 

**Table 2:** Ex vivo biodistribution of [^18^F]FET after intravenous injection in athymic nu/nu male mice HCT116 xenografts.

**Organs**	**5 min (n=5**)			**30 min (n=4**)			**60 min (n=4**)		
blood	3.89	+/-	0.41	4.13	+/-	0.25	4.20	+/-	0.71
lung	3.41	+/-	0.49	3.45	+/-	0.19	3.90	+/-	0.60
liver(all)	3.17	+/-	0.43	3.11	+/-	0.19	3.69	+/-	0.67
spleen	3.39	+/-	0.66	3.18	+/-	0.18	3.07	+/-	0.59
kidney	3.57	+/-	0.46	3.17	+/-	0.22	4.38	+/-	0.47
muscle	2.95	+/-	0.64	3.17	+/-	0.31	3.46	+/-	0.59
heart	3.25	+/-	0.66	3.60	+/-	0.14	3.72	+/-	0.63
brain	2.38	+/-	0.34	2.64	+/-	0.14	2.63	+/-	0.39
bone	1.52	+/-	0.24	2.14	+/-	0.30	1.55	+/-	0.25
testes	2.13	+/-	0.45	2.37	+/-	0.23	2.73	+/-	0.51
pancreas	9.83	+/-	2.86	11.52	+/-	2.26	11.29	+/-	2.50
tumor	2.75	+/-	0.69	4.38	+/-	0.51	3.17	+/-	0.72
stomach	2.69	+/-	0.93	2.37	+/-	0.35	2.88	+/-	1.48
sm int	3.26	+/-	0.54	3.48	+/-	0.19	3.81	+/-	0.76
u lg int	2.85	+/-	0.48	3.00	+/-	0.18	3.33	+/-	0.49
l lg int	2.23	+/-	0.29	4.59	+/-	0.39	2.75	+/-	0.45
Data expressed in %ID/g ± SD									

## Discussion

The system L transporter, LAT1, is an emerging biomarker for tumor aggressiveness and prognosis. Additionally, this amino acid transporter is being explored for targeted delivery of therapeutic agents. To date approaches to non-invasively image LAT1 transporter levels in malignant tissue have focused on radiolabeled amino acids that are transport substrates for LAT1. However, these substrates have limited utility outside of the brain due to relatively low sensitivity for tumor. This limitation led us to develop an immunoPET agent targeting amino acid transporters. The novel ^89^Zr-labeled anti-LAT mAb, [^89^Zr]DFO-Ab2, demonstrates high affinity and specificity for human LAT1 *in vitro* and *in vivo*. Flow cytometry data using Ab2 shows no cross-reactivity with other heterodimeric amino acid transporters including the system L transporter LAT2 (see Figure S1). Incorporation of metal chelator, DFO, does not significantly affect the immunoreactivity and provides a means for high labeling efficiency with ^89^Zr which has physical half-life (3.3 days) compatible with the pharmacokinetics of intact mAbs. Biodistribution and small animal PET/CT studies with [^89^Zr]DFO-Ab2 show *in vivo* binding to LAT1 in the HCT116 colorectal cancer model, a cell line that is known to express LAT1 in high abundance [22]. Tumor uptake of the tracer was high and persistent with tumor associated activity of 9.3 ± 0.84 and 10.5 ± 1.8 %ID/g at 3 and 7 d p.i., respectively. Excellent tumor-to-non-target organ contrast was achieved at 7 days with tumor-to-blood and tumor-to-muscle ratios of 3.5:1 and 13:1 respectively compared to 1.1:1 and 1.4:1 for the system L transport substrate, [^18^F]FET, at 30 minutes. The results strongly suggest the superiority of immunoPET targeting LAT1 over system L transport substrates like [^18^F]FET for neoplasms located outside of the brain. A number of radiolabeled amino acids targeting system L transport including [^123^I]IMT , [^18^F]FDOPA, and [^18^F]FAMT have been evaluated in humans for oncologic imaging. The presence of an α-methyl group increases the selectivity of aromatic amino acids for LAT1 over other system L transporters, and tracers like IMT and FAMT may show somewhat higher tumor to background ratios compared to FET [17]. However, the exchange mechanism of LAT1 transport limits the tumor to background ratios that can be achieved with substrate-based imaging agents. 

While the [^89^Zr]DFO-Ab2 has superior contrast in all organs compared to [^18^F]FET, the low normal tissue uptake of [^89^Zr]DFO-Ab2 in mice may be due in part to lack of recognition of mouse LAT1 by this mAb (Figure S1). Ab2 is an anti-human LAT1 antibody with no cross species reactivity, and thus LAT1 binding in normal mouse tissues does not occur, as confirmed by the blocking study where all organ distribution with exception of the tumor were similar (see Table 1). The lack of binding to LAT1 in normal tissues may artificially increase the tumor to tissue ratios observed in these studies. *In vivo* characterization of amino acid transporter substrates are also limited as the relative transport rates of [^18^F]FET by human LAT1 versus its mouse homologue have not yet been measured. The species differences may contribute to the differences in [^18^F]FET uptake by human tumor xenografts and normal mouse tissues, however, [^18^F]FET has been successfully used to image tumors derived from mouse and rat cell lines, indicating this tracer is recognized by rodent system L transporters [35-37]. LAT1 is regarded as an oncofetal protein and its’ expression is limited in normal adult tissue, thus, the uptake of [^89^Zr]DFO-Ab2 in most human tissues and organs may be relatively low as in this mouse model. The high liver uptake of [^89^Zr]DFO-Ab2 is possibly a result of the biological clearance of the tracer, and is a profile typical of radiolabeled antibodies. However, colorectal carcinoma frequently metastasizes to the liver, and the low tumor to liver contrast of 1.1:1 may limit the utility of the [^89^Zr]DFO-Ab2 for detecting lesions in the liver. Despite the potential limitations, our results collectively show immunoPET has potential advantages including higher specificity for LAT1 and improved tumor visualization compared to the traditional substrate-based approach. The reported immunoPET agent, [^89^Zr]DFO-Ab2, is the first example of a macromolecule against LAT1. Other constructs such as affibodies or minibodies may also be advantageous techniques for directly probing expression of LAT1. Currently, these approaches have yet to be explored; however, the availability of effective non-substrate tracers for imaging LAT1 would enable detailed investigations into the relative importance of LAT1 protein levels versus substrate flux in tumor biology.

A potential confound when evaluating large-molecule probes like antibodies is enhanced permeability and retention (EPR) effect due to the leaky vasculature characteristic of solid tumors. To confirm that the accumulation of [^89^Zr]DFO-Ab2 in tumor tissue was due to specific binding rather than non-specific mechanisms, control experiments using non-specific polyclonal ^89^Zr-labeled IgG and a blocking study using excess unlabeled Ab2 were employed. We observed 5-fold higher tumor uptake with [^89^Zr]DFO-Ab2 compared to non-specific rat IgG and a 55 % decrease in tumor associated activity with preinjection of a 50-fold excess by weight of unmodified antibody. Together with the *in vitro* data, these experiments indicate that [^89^Zr]DFO-Ab2 binds specifically to LAT1 *in vivo*.

Radiolabeled amino acids such as [^18^F]FET have been most successfully utilized clinically for brain tumors but have been less effective for extracranial malignancies. System L is active at the blood-brain barrier (BBB) which allows transport substrates to reach the entire brain tumor volume including non-contrast enhancing regions on magnetic resonance imaging (MRI) without disrupted BBB. Additional studies will be needed to assess the utility of [^89^Zr]DFO-Ab2 for imaging brain tumors that do not exhibit contrast enhancement due to intact BBB. It is possible that LAT1 upregulation in the endothelium of non-enhancing gliomas could serve as an imaging target for [^89^Zr]DFO-Ab2 and related compounds. Alternatively, receptor-mediated transcytosis approaches that exploit the transferrin and insulin-like growth factor receptors for targeted delivery of large antibodies and nanoparticles in mouse, rat, and Rhesus monkey models could be used [38-41]. Further work will be needed to elucidate the role of immunoPET targeting LAT1 in brain tumors that do not exhibit contrast enhancement due to intact BBB. 

In conclusion, we have developed an immunoPET agent targeting human LAT1 that demonstrates specific in vivo binding in a mouse model of colorectal cancer with excellent tumor visualization. To our knowledge, these results are the first reported example of a PET imaging agent that directly binds to a specific amino acid transporter. Given the importance of LAT1 in human cancer, the ability to directly visualize and quantify LAT1 transporter density has potential utility as a new tool for oncologic imaging. This imaging strategy also has the potential to substantially extend amino acid transporter-based oncologic imaging to a wide range of neoplasms not effectively imaged with currently available radiolabeled amino acids. 

## Supporting Information

Figure S1 **Ab2 reactivity with LAT1 in RH7777 cells transfected with cDNA of green fluorescent protein (GFP) fused human CD98hc or various human CD98lcs.**
(TIF)

## References

[1] Ohno Y, Suda K, Masuko K, Yagi H, Hashimoto Y et al. (2008) Production and characterization of highly tumor-specific rat monoclonal antibodies recognizing the extracellular domain of human L-type amino-acid transporter 1. Cancer Sci 99: 1000-1007. doi:10.1111/j.1349-7006.2008.00770.x. PubMed: 18294274. 18294274 PMC11160021

[2] Fuchs BC, Bode BP (2005) Amino acid transporters ASCT2 and LAT1 in cancer: partners in crime? Semin Cancer Biol 15: 254-266. doi:10.1016/j.semcancer.2005.04.005. PubMed: 15916903. 15916903

[3] Kanai Y, Segawa H, Miyamoto KI, Uchino H, Takeda E et al. (1998) Expression Cloning and Characterization of a Transporter for Large Neutral Amino Acids Activated by the Heavy Chain of 4F2 Antigen (CD98). J Biol Chem 273: 23629-23632. doi:10.1074/jbc.273.37.23629. PubMed: 9726963. 9726963

[4] Teixeira S, Di Grandi S, Kühn LC (1987) Primary structure of the human 4F2 antigen heavy chain predicts a transmembrane protein with a cytoplasmic NH2 terminus. J Biol Chem 262: 9574-9580. PubMed: 3036867. 3036867

[5] Yanagida O, Kanai Y, Chairoungdua A, Kim DK, Segawa H et al. (2001) Human L-type amino acid transporter 1 (LAT1): characterization of function and expression in tumor cell lines. Biochimica et Biophysica Acta (BBA) - Biomembranes 1514: 291-302. doi:10.1016/S0005-2736(01)00384-4. PubMed: 11557028. 11557028

[6] Jewell JL, Guan KL (2013) Nutrient signaling to mTOR and cell growth. Trends Biochem Sci, 38: 233–42. PubMed: 23465396. 23465396 10.1016/j.tibs.2013.01.004 PMC3634910

[7] Fan X, Ross DD, Arakawa H, Ganapathy V, Tamai I et al. (2010) Impact of system L amino acid transporter 1 (LAT1) on proliferation of human ovarian cancer cells: a possible target for combination therapy with anti-proliferative aminopeptidase inhibitors. Biochem Pharmacol 80: 811-818. doi:10.1016/j.bcp.2010.05.021. PubMed: 20510678. 20510678

[8] Nicklin P, Bergman P, Zhang B, Triantafellow E, Wang H et al. (2009) Bidirectional transport of amino acids regulates mTOR and autophagy. Cell 136: 521-534. doi:10.1016/j.cell.2008.11.044. PubMed: 19203585. 19203585 PMC3733119

[9] Kaira K, Oriuchi N, Imai H, Shimizu K, Yanagitani N et al. (2009) Prognostic significance of L-type amino acid transporter 1 (LAT1) and 4F2 heavy chain (CD98) expression in early stage squamous cell carcinoma of the lung. Cancer Sci 100: 248-254. PubMed: 19068093. 19068093 10.1111/j.1349-7006.2008.01029.x PMC11159214

[10] Kaira K, Oriuchi N, Imai H, Shimizu K, Yanagitani N et al. (2008) Prognostic significance of L-type amino acid transporter 1 expression in resectable stage I-III nonsmall cell lung cancer. Br J Cancer 98: 742-748. doi:10.1038/sj.bjc.6604235. PubMed: 18253116. 18253116 PMC2259171

[11] Sakata T, Ferdous G, Tsuruta T, Satoh T, Baba S et al. (2009) L-type amino-acid transporter 1 as a novel biomarker for high-grade malignancy in prostate cancer. Pathol Int 59: 7-18. doi:10.1111/j.1440-1827.2008.02319.x. PubMed: 19121087. 19121087

[12] Nawashiro H, Otani N, Shinomiya N, Fukui S, Ooigawa H et al. (2006) L-type amino acid transporter 1 as a potential molecular target in human astrocytic tumors. Int J Cancer 119: 484-492. doi:10.1002/ijc.21866. PubMed: 16496379. 16496379

[13] Chen W, Silverman DH, Delaloye S, Czernin J, Kamdar N et al. (2006) F-FDOPA PET imaging of brain tumors: comparison study with 18 F-FDG PET and evaluation of diagnostic accuracy 18. J Nucl Med 47: 904-911. PubMed: 16741298. 16741298

[14] Pauleit D, Stoffels G, Bachofner A, Floeth FW, Sabel M et al. (2009) Comparison of (18)F-FET and (18)F-FDG PET in brain tumors. Nucl Med Biol 36: 779-787. doi:10.1016/j.nucmedbio.2009.05.005. PubMed: 19720290. 19720290

[15] Pauleit D, Floeth F, Tellmann L, Hamacher K, Hautzel H et al. (2004) Comparison of *O*-(2-^18^F-fluoroethyl)-L-tyrosine PET and 3-^123^I-iodo-alpha-methyl-L-tyrosine SPECT in brain tumors. J Nucl Med 45: 374-381. PubMed: 15001676. 15001676

[16] Grosu AL, Astner ST, Riedel E, Nieder C, Wiedenmann N et al. (2011) An interindividual comparison of O-(2-[18F]fluoroethyl)-L-tyrosine (FET)- and L-[methyl-11C]methionine (MET)-PET in patients with brain gliomas and metastases. Int J Radiat Oncol Biol Phys 81: 1049-1058. doi:10.1016/j.ijrobp.2010.07.002. PubMed: 21570201. 21570201

[17] Watanabe H, Inoue T, Shinozaki T, Yanagawa T, Ahmed AR et al. (2000) PET imaging of musculoskeletal tumours with fluorine-18 alpha-methyltyrosine: comparison with fluorine-18 fluorodeoxyglucose PET. Eur J Nucl Med 27: 1509-1517. doi:10.1007/s002590000344. PubMed: 11083540. 11083540

[18] Jager PL, Vaalburg W, Pruim J, de Vries EG, Langen KJ et al. (2001) Radiolabeled amino acids: basic aspects and clinical applications in oncology. J Nucl Med 42: 432-445. PubMed: 11337520. 11337520

[19] Youland RS, Kitange GJ, Peterson TE, Pafundi DH, Ramiscal JA et al. (2013) The role of LAT1 in (18)F-DOPA uptake in malignant gliomas. J Neuro Oncol 111: 11-18. doi:10.1007/s11060-012-0986-1. PubMed: 23086431. PMC3907171 23086431

[20] Lahoutte T, Caveliers V, Camargo SM, Franca R, Ramadan T et al. (2004) SPECT and PET amino acid tracer influx via system L (h4F2hc-hLAT1) and its transstimulation. J Nucl Med 45: 1591-1596. PubMed: 15347729. 15347729

[21] Haase C, Bergmann R, Fuechtner F, Hoepping A, Pietzsch J (2007) L-Type Amino Acid Transporters LAT1 and LAT4 in Cancer: Uptake of 3-O-Methyl-6- 18F-Fluoro-l-Dopa in Human Adenocarcinoma and Squamous Cell Carcinoma In Vitro and In Vivo. J Nucl Med 48: 2063-2071. doi:10.2967/jnumed.107.043620. PubMed: 18056335. 18056335

[22] Ohkawa M, Ohno Y, Masuko K, Takeuchi A, Suda K et al. (2011) Oncogenicity of L-type amino-acid transporter 1 (LAT1) revealed by targeted gene disruption in chicken DT40 cells: LAT1 is a promising molecular target for human cancer therapy. Biochem Biophys Res Commun 406: 649-655. doi:10.1016/j.bbrc.2011.02.135. PubMed: 21371427. 21371427

[23] Masuko T, Ohno Y, Masuko K, Yagi H, Uejima S et al. (2011) Towards therapeutic antibodies to membrane oncoproteins by a robust strategy using rats immunized with transfectants expressing target molecules fused to green fluorescent protein. Cancer Sci 102: 25-35. doi:10.1111/j.1349-7006.2010.01741.x. PubMed: 21040216. 21040216

[24] Vosjan MJ, Perk LR, Visser GW, Budde M, Jurek P et al. (2010) Conjugation and radiolabeling of monoclonal antibodies with zirconium-89 for PET imaging using the bifunctional chelate p-isothiocyanatobenzyl-desferrioxamine. Nat Protoc 5: 739-743. doi:10.1038/nprot.2010.13. PubMed: 20360768. 20360768

[25] Holland JP, Divilov V, Bander NH, Smith-Jones PM, Larson SM et al. (2010) 89Zr-DFO-J591 for immunoPET of prostate-specific membrane antigen expression in vivo. J Nucl Med 51: 1293-1300. doi:10.2967/jnumed.110.076174. PubMed: 20660376. 20660376 PMC2998794

[26] Dijkers EC, Oude Munnink TH, Kosterink JG, Brouwers AH, Jager PL et al. (2010) Biodistribution of 89Zr-trastuzumab and PET imaging of HER2-positive lesions in patients with metastatic breast cancer. Clin Pharmacol Ther 87: 586-592. doi:10.1038/clpt.2010.12. PubMed: 20357763. 20357763

[27] Chang AJ, De Silva RA, Lapi SE (2013) Development and characterization of 89Zr-labeled panitumumab for immuno-positron emission tomographic imaging of the epidermal growth factor receptor. Mol Imaging 12: 17-27. PubMed: 23348788. 23348788 PMC4329987

[28] Holland JP, Sheh Y, Lewis JS (2009) Standardized methods for the production of high specific-activity zirconium-89. Nucl Med Biol 36: 729-739. doi:10.1016/j.nucmedbio.2009.05.007. PubMed: 19720285. 19720285 PMC2827875

[29] Zuhayra M, Alfteimi A, Forstner CV, Lützen U, Meller B et al. (2009) New approach for the synthesis of [18F]fluoroethyltyrosine for cancer imaging: Simple, fast, and high yielding automated synthesis. Bioorganic &amp. J Med Chem 17: 7441-7448. 10.1016/j.bmc.2009.09.029 19804977

[30] Lindmo T, Boven E, Cuttitta F, Fedorko J, Bunn PA Jr. (1984) Determination of the immunoreactive fraction of radiolabeled monoclonal antibodies by linear extrapolation to binding at infinite antigen excess. J Immunol Methods 72: 77-89. doi:10.1016/0022-1759(84)90435-6. PubMed: 6086763. 6086763

[31] Rudnick SI, Lou J, Shaller CC, Tang Y, Klein-Szanto AJP et al. (2011) Influence of Affinity and Antigen Internalization on the Uptake and Penetration of Anti-HER2 Antibodies in Solid Tumors. Cancer Res 71: 2250-2259. doi:10.1158/0008-5472.CAN-10-2277. PubMed: 21406401. 21406401 PMC3077882

[32] Dijkers EC, Kosterink JG, Rademaker AP, Perk LR, van Dongen GA et al. (2009) Development and characterization of clinical-grade 89Zr-trastuzumab for HER2/neu immunoPET imaging. J Nucl Med 50: 974-981. doi:10.2967/jnumed.108.060392. PubMed: 19443585. 19443585

[33] Nayak TK, Garmestani K, Milenic DE, Brechbiel MW (2012) PET and MRI of metastatic peritoneal and pulmonary colorectal cancer in mice with human epidermal growth factor receptor 1-targeted 89Zr-labeled panitumumab. J Nucl Med 53: 113-120. doi:10.2967/jnumed.111.094169. PubMed: 22213822. 22213822 PMC3252203

[34] Abou DS, Ku T, Smith-Jones PM (2011) In vivo biodistribution and accumulation of 89Zr in mice. Nucl Med Biol 38: 675-681. doi:10.1016/j.nucmedbio.2010.12.011. PubMed: 21718943. 21718943 PMC4527328

[35] Solingapuram Sai KK, Huang C, Yuan L, Zhou D, Piwnica-Worms D et al. (2013) 18F-AFETP, 18F-FET, and 18F-FDG Imaging of Mouse DBT Gliomas. J Nucl Med 54: 1120-1126. doi:10.2967/jnumed.112.113217. PubMed: 23650628. 23650628 PMC3766958

[36] Lee TS, Ahn SH, Moon BS, Chun KS, Kang JH et al. (2009) Comparison of 18F-FDG, 18F-FET and 18F-FLT for differentiation between tumor and inflammation in rats. Nucl Med Biol 36: 681-686. doi:10.1016/j.nucmedbio.2009.03.009. PubMed: 19647174. 19647174

[37] Spaeth N, Wyss MT, Weber B, Scheidegger S, Lutz A et al. (2004) Uptake of 18F-fluorocholine, 18F-fluoroethyl-L-tyrosine, and 18F-FDG in acute cerebral radiation injury in the rat: implications for separation of radiation necrosis from tumor recurrence. J Nucl Med 45: 1931-1938. PubMed: 15534065. 15534065

[38] Jones AR, Shusta EV (2007) Blood-brain barrier transport of therapeutics via receptor-mediation. Pharm Res 24: 1759-1771. doi:10.1007/s11095-007-9379-0. PubMed: 17619996. 17619996 PMC2685177

[39] Yu YJ, Zhang Y, Kenrick M, Hoyte K, Luk W et al. (2011) Boosting brain uptake of a therapeutic antibody by reducing its affinity for a transcytosis target. Sci Transl Med 3: 84ra44 PubMed: 21613623. 10.1126/scitranslmed.3002230 21613623

[40] Boado RJ, Zhang Y, Pardridge WM (2007) Genetic engineering, expression, and activity of a fusion protein of a human neurotrophin and a molecular Trojan horse for delivery across the human blood-brain barrier. Biotechnol Bioeng 97: 1376-1386. doi:10.1002/bit.21369. PubMed: 17286273. 17286273

[41] Mishra V, Mahor S, Rawat A, Gupta PN, Dubey P et al. (2006) Targeted brain delivery of AZT via transferrin anchored pegylated albumin nanoparticles. J Drug Target 14: 45-53. doi:10.1080/10611860600612953. PubMed: 16603451. 16603451

