# Supplementary figures and images for: Imaging the L-Type Amino Acid Transporter-1 (LAT1) with Zr-89 ImmunoPET
Source: PLoS One. 2013 Oct 15;8(10):e77476. doi: 10.1371/journal.pone.0077476 (PMC3797081; doi:10.1371/journal.pone.0077476)

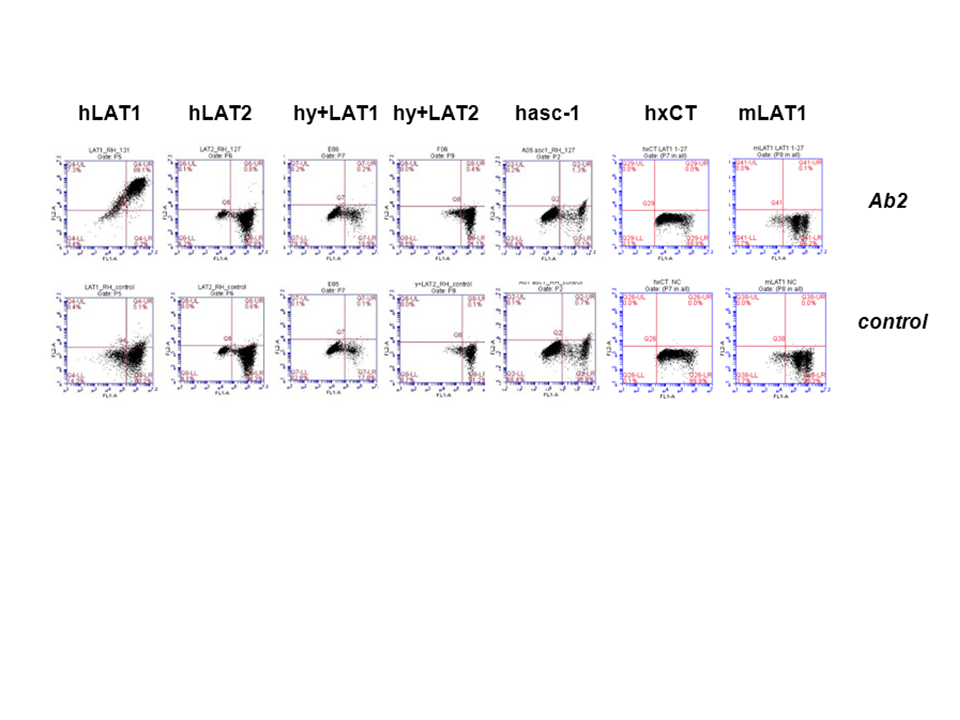

Supplement: Figure S1 — Ab2 reactivity with LAT1 in RH7777 cells transfected with cDNA of green fluorescent protein (GFP) fused human CD98hc or various human CD98lcs. (TIF) [file pone.0077476.s001.tif]
